# Off-label MRI examination of an MR conditional pacemaker

**DOI:** 10.1259/bjrcr.20150219

**Published:** 2016-05-15

**Authors:** Jonathon Delve, Peter Wright, Ashish Patwala, Dipti Patel, Matthew Stevenson

**Affiliations:** ^1^ Radiology Physics, University Hospitals of North Midlands NHS Trust, Royal Stroke University Hospital, Stroke on Trent, UK; ^2^ Medical Physics and Medical Imaging, Sheffield Teaching Hospitals NHS Foundation Trust, Sheffield, UK; ^3^ Department of Cardiology, University Hospitals of North Midlands NHS Trust, Royal Stoke University Hospital, Stroke on Trent, UK; ^4^ Rheumatology Department, Haywood Hospital, Stoke on Trent, UK

## Abstract

An 18-year-old patient was implanted with an MR-conditional pacemaker using a lead plug which negated the manufacturer CE approved MR conditional status of the pacing system. The patient was referred for a lumbar spine MRI. The clinical opinion was that the risk of proceeding with the MRI was acceptable given the alternatives of: (a) changing the pacing system or (b) not performing the scan. A risk assessment revealed that the additional risk was very low and easily mitigated by an established technique. Informed consent was obtained and the scan was completed without incident following a slightly modified MR-conditional pacemaker scanning protocol.

## Introduction

The population of patients fitted with pacemakers and implantable cardiac defibrillators grows year on year, as does the proportion of that demographic fitted with MR-conditional devices. At University Hospital of North Midlands (UHNS), the vast majority of active cardiac devices implanted are MR conditional. Cases overcoming complications regarding MRI examinations involving these devices are therefore of increasing significance.

Pacemaker systems rated as MR conditional are certified as such after extensive testing by the manufacturer. Among other requirements for the safe scanning of these devices, implanted systems must conform to the list of pulse generator and lead combinations tested to retain the MR conditional status. This report describes the successful MRI examination of a patient with a pacemaker system that differed from its sanctioned MR conditional configuration.

## Presentation

An 18-year-old female was referred for a lumbar spine MRI examination at UHNS. She had had a pacemaker system (Medtronic Ensura DR MRI, Medtronic Inc., Minneapolis, MN) implanted 2 months previously to treat Mobitz Type 2 block, but with only the ventricular lead (Medtronic CapSureFix Novus MRI SureScan) implanted. The atrial lead port was closed with a port plug. This was carried out to reduce the complexity of device removal and replacement later in life. However, this configuration of pulse generator, single lead and plug was not included in the manufacturer’s list of MR conditional configurations^1^ and therefore any MRI examination would be an off-label use of the device.

Alternative imaging (*e.g.* low-dose CT scan) had been considered and rejected as imaging was requested via a neurology referral for the lumber spine and the spinal cord. The opinion of cardiology was sought regarding the device. Their judgement was that the risks of performing the MRI were less than those associated with changing the pacing system to fit an established MR conditional configuration.

## Investigation

As per Medicines and Healthcare products Regulatory Agency (MHRA) guidelines for the off-label use of a medical device,^2^ MR physics conducted a risk assessment to establish the severity of any additional risks from the use of the device and formulate preventative measures to mitigate these risks where possible (Table 1).

**Table 1.** Risk assessment for off-label use of pacing system

Hazard	Cause	Comparison to risk for complete SureScan system	Additional precautions above Medtronic safety instructions
Mechanical stress/displacement	Static magnetic field; incomplete fibrosis	Reduced due to fewer components	None: risk already controlled
Induced stimulation	Gradient fields; RF absorption; ohmic heating	Comparable	None: risk already controlled
Discomfort and/or burns	RF absorption; ohmic heating	Potentially increased due to fewer alternative current paths	See investigation
Image artefacts	Magnetic inhomogeneity	Reduced due to fewer components	None: risk already controlled
Altered pacing	Gradient fields, RF absorption	Increased owing to inoperability of MR safe mode	See investigation

RF, radiofrequency.

The risk of damage from heating is increased with increasing radiofrequency (RF) power deposition and RF emission duration. These are considered in specific absorption rate (SAR) calculations included in every scan sequence for the Siemens 1.5 T Aera system (Siemens Healthcare GmbH, Erlangen, Germany) used. Therefore SAR limits can be used to reduce this risk. Haemodynamic monitoring required under the manufacturer’s guidelines for the scanning of SureScan systems would identify any alteration in cardiac function due to heating or heat-induced tissue damage.

Examination of records for previous patients of similar build, age and sex undergoing the same examination showed that the SAR for the sequences to be run did not exceed a per-sequence average of 1.74 W kg^–1^. This is 13% below the SAR limit required for the scanning of SureScan systems, and demonstrates that any potential heating of the device is unlikely to cause damage during sequence run-time. Additional risk mitigation was achieved by leaving rest periods between sequences to allow physiological thermoregulatory systems to normalize the temperature of the pacing system and surrounding tissue should any heating have occurred.

The replacement of one lead with a port plug meant that no impedance measurement could be obtained across that port, meaning that the MR mode could not be activated. However, as the patient was not pacing dependent, this did not constitute an additional risk.

## Outcome

Informed consent was obtained from the patient by the supervising cardiologist. The patient's pacemaker device was interrogated prior to MRI to confirm that the ventricular lead was within threshold tolerances and was scanned under the normal MR conditional pacemaker protocol. This incorporated the manufacturer’s guidelines and the inclusion of longer rest periods between sequences. The MRI examination was performed with no ill effects. Diagnostic images were obtained and no damage to the pacing system was detected on post-MRI interrogation of the device. Communication between the MR radiographer and the patient was maintained throughout the MR scan and the patient reported no discomfort or adverse heating. Normal function of the pacing system was verified after the MRI examination.

## Conclusions

This case demonstrates that MRI scanning at 1.5 T may be carried out safely for this system configuration after suitable risk assessment and actioning suitable risk mitigation measures. The MHRA guidelines regarding the off-label use of medical devices applicable to MR-conditional pacemaker systems appear appropriate for assessment of contraindication and in identifying risk minimization procedures.

## Learning points

1. While the above-described system configuration is contraindicated under the manufacturer’s MRI instructions, scanning may be performed without incident after a suitable risk/benefit assessment and risk mitigation measures are put in place.
2. Medtronic SureScan pacemakers cannot enter MR mode if one or more ports are plugged and as such, this system configuration remains contraindicated for any pacing modes where this would be required.

## Consent

Informed consent to publish this case report was obtained from the patient in line with the *BJR*|*case report* consent policy.
